# Incidence of Acute Kidney Injury in Autologous Hematopoietic Stem Cell Transplant Recipients According to the Administration of Empirical Amikacin: A Two-Centre Retrospective Cohort Study

**DOI:** 10.3390/antibiotics14090919

**Published:** 2025-09-11

**Authors:** Sophie Schürch, Sarah Dräger, Michèle Hoffmann, Severin Bausch, Nicolas Gürtler, Cédric Hirzel, Jakob Passweg, Stefano Bassetti, Thomas Pabst, Parham Sendi, Michael Osthoff

**Affiliations:** 1 Division of Internal Medicine, University Hospital Basel, 4031 Basel, Switzerland; 2 Department of Clinical Research, University of Basel, 4031 Basel, Switzerland; 3 Department of Medical Oncology, Bern University Hospital, University of Bern, 3010 Bern, Switzerland; 4 Department of Infectious Diseases, Bern University Hospital, University of Bern, 3010 Bern, Switzerland; 5 Division of Hematology, Department of Medicine, University Hospital Basel, University of Basel, 4031 Basel, Switzerland; 6 Institute for Infectious Diseases, University of Bern, 3012 Bern, Switzerland; 7 Department of Internal Medicine, Cantonal Hospital Winterthur, 8400 Winterthur, Switzerland

**Keywords:** acute kidney injury, autologous hematopoietic stem cell transplantation, empirical antibiotic treatment, beta-lactam antibiotic, amikacin

## Abstract

**Background**: The benefit of adjunctive aminoglycosides in the treatment of patients with febrile neutropenia (FN) is controversial. We investigated the incidence of acute kidney injury (AKI) in patients with FN or suspected infection according to empirical amikacin treatment. **Methods**: This two-centre, retrospective cohort study was conducted at the University Hospitals of Basel (amikacin group) and Bern (non-amikacin group), Switzerland, between 2016 and 2022. Adult patients requiring antibiotic treatment after autologous hematopoietic stem cell transplantation (HSCT) were included. All patients received empiric beta-lactam treatment combined with amikacin in the amikacin group (only University Hospital Basel). The primary endpoint was the incidence of AKI within seven days after the initiation of antibiotic treatment. **Results**: Overall, 250 patients were included. The majority was male (n = 163, 65.2%) and had a median age of 61 years (interquartile range (IQR) 55 to 67). The median baseline eGFR was similar in both groups (>90 mL/min/1.7 m^2^). There was no statistically significant difference in the incidence of AKI (4/125 vs. 5/125, *p* = 1.0). The maximum decline in eGFR from baseline within 7 days was significantly higher in the amikacin group (−4 mL/min/1.7 m^2^ (IQR 8 to −12) vs. −2 mL/min/1.7 m^2^ (IQR −7 to −1), *p* = 0.001). Two patients suffered from an infection with an extended spectrum beta-lactamase producing (ESBL) pathogen. **Conclusions**: Amikacin treatment did not significantly impact the incidence of AKI in patients undergoing autologous HSCT. The short-term administration of amikacin in patients with normal to high baseline eGFR is safe regarding renal function. However, in a low-resistance setting, the omission of empirical amikacin treatment should be considered.

## 1. Introduction

Autologous hematopoietic stem-cell transplantation (HSCT) is a standard treatment option for haematological disorders such as multiple myeloma or (Non)-Hodgkin’s lymphoma. HSCT recipients are at high risk of developing febrile neutropenia (FN) or severe infection [1,2], which requires the immediate initiation of empirical broad-spectrum antibiotic treatment [3].

Two empirical antibiotic treatment strategies for patients with FN are recommended: a de-escalation or an escalation strategy [4,5,6]. The de-escalation strategy includes a carbapenem as monotherapy or a combination therapy of an anti-pseudomonal beta-lactam combined with an aminoglycoside, such as gentamicin, tobramycin, or amikacin [4,7,8]. Conversely, the escalation strategy consists of monotherapy with an anti-pseudomonal beta-lactam like cefepime or piperacillin/tazobactam [9,10]. The choice of the optimal empirical antibiotic treatment regimen depends on the local epidemiology and may vary between different countries and hospitals [11,12].

Amikacin is one of the aminoglycosides used in the de-escalation approach. It exerts a rapid bactericidal effect against many aerobic gram-negative pathogens [13]. A common side effect is dose-dependent acute kidney injury (AKI), particularly with prolonged administration, as the drug accumulates in the proximal renal tubular cells [14]. High plasma trough concentration, older age, prolonged treatment, liver dysfunction, and hypoalbuminemia are associated with increased nephrotoxicity [15,16,17].

Data regarding the nephrotoxic side effects of amikacin in patients treated for FN or severe infection after autologous HSCT are limited. In addition, recent data suggest that higher dosages of amikacin are required for target attainment [18]. However, patients undergoing autologous HSCT for multiple myeloma often suffer from renal impairment due to various reasons such as the deposition of amyloid or light chains in the kidneys, hypercalcemia, treatment-related nephrotoxicity, or cast nephropathy. Therefore, the benefit of enhanced coverage against multidrug-resistant pathogens with the addition of an aminoglycoside should be carefully balanced against the risk of kidney injury. The aim of this study is to compare the incidence of AKI in autologous HSCT recipients receiving empirical antibiotic treatment with or without amikacin.

## 2. Results

### 2.1. Patient Characteristics

Overall, out of 337 patients, 250 patients (125 patients from each centre) were included in the study (Supplementary Materials, Figure S1). Baseline characteristics and clinical outcomes were similar in the two groups, except for the Charlson Comorbidity Score, the leucocyte count, the albumin, the site of infection, and the length of stay, which were significantly different between the two groups. However, the absolute differences were usually small (Table 1).

The majority of patients were male (n = 163, 65.2%) and the median age was 61 years (IQR 55−67). Multiple myeloma was the most frequent underlying haematological disorder (n = 137, 54.8%), followed by lymphoma (n = 97, 38.8%). The gastrointestinal tract, including mucositis, was the most frequent site of infection (n = 166/250, 66.4%). In 26.8% (n = 67/250), bloodstream infection was present. Two patients (non-amikacin group) had an infection due to an ESBL-producing *Escherichia coli* (urinary tract infection and bloodstream infection) and five patients were known to be colonised with an ESBL-producing pathogen (n = 3 in the amikacin-group and n = 2 in the non-amikacin group, respectively). All ESBL-producing pathogens in the amikacin group were tested to be susceptible to amikacin.

### 2.2. Renal Function and AKI

**Figure 1:**
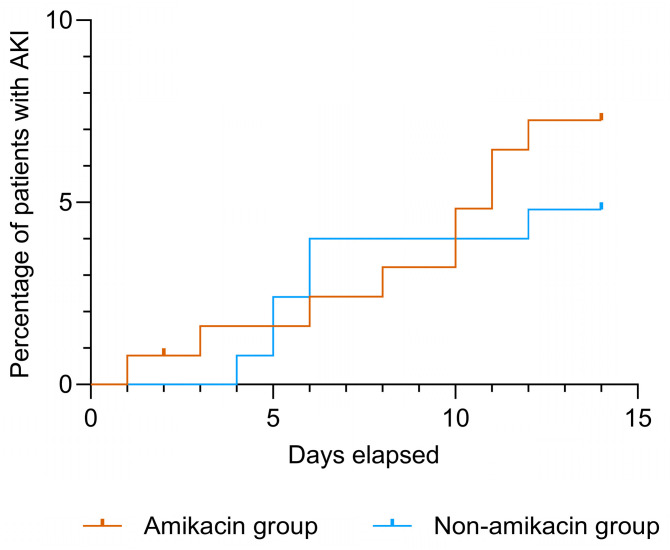
Incidence of acute kidney injury (AKI) within 14 days according to the administration of amikacin.

The median baseline eGFR at the initiation of antibiotic treatment was 96 mL/min/1.7 m^2^ (IQR 85−105) in the amikacin group and 100 mL/min/1.7 m^2^ (IQR 86−111) in the non-amikacin group (*p* = 0.06). An eGFR of <60 mL/min/1.7 m^2^ was evident at baseline in nine patients in the amikacin group and in 12 patients in the non-amikacin group, where one had an eGFR of <30 mL/min/1.7 m^2^. The incidence of AKI within seven days after the initiation of antibiotic treatment was 3.2% (n = 4) in the amikacin group and 4.0% (n = 5) in the non-amikacin group (*p* = 1.0) (Figure 1). Only 1 of the 21 patients with a baseline eGFR ˂ 60 mL/min/1.7 m^2^ developed AKI.

All patients (n = 9) with AKI developed AKI stage 1. The course of eGFR according to the development of AKI is depicted in the Supplement, Figure S2. Renal function recovered in 88.8% (n = 8/9) of the patients within 90 days. One patient with AKI died within 30 days (non-amikacin group). Patients who developed AKI were significantly older (median 68 years (IQR 63−70) vs. 61 years (IQR 55−67), *p* = 0.043) and had a lower median eGFR at baseline (84 mL/min/1.7 m^2^ (IQR 78−93) vs. 98 mL/min/1.7 m^2^ (IQR 86−108), *p* = 0.08). Eight out of the nine patients received at least one concomitant, potentially nephrotoxic drug (diuretics).

All patients who developed AKI in the amikacin group had an amikacin treatment duration of at least two days. There was no difference in the median first amikacin dose between patients who developed AKI and those who did not (13.8 mg/kg/of body weight vs. 12.1 mg/kg of body weight, *p* = 0.43) (Supplementary Materials, Figure S3). One patient who developed AKI did not receive the appropriate amikacin dose on the first day of amikacin treatment (administered dose lower than recommended, 1000 mg vs. 1200 mg), whereas the other three patients were dosed appropriately. Two of the patients who developed AKI were obese; however, they were dosed appropriately. There was no statistically significant difference in the cumulative amikacin dose between patients who were suffering from AKI and those who were not (2500 mg vs. 2250 mg, *p* = 0.39) (Supplementary Materials, Figure S4).

**Figure 2:**
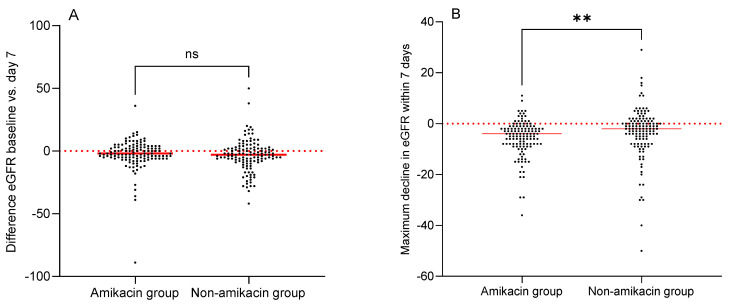
Difference between baseline eGFR and eGFR at day 7 (**A**) and maximum decline in eGFR within the first seven days (**B**). Medians are depicted with a red horizontal line. ** statistically significant. The red dotted line represents the zero line. Abbreviations: eGFR, estimated glomerular filtration rate; ns, not statistically significant.

The median absolute difference between the eGFR at day 7 and the baseline eGFR was −2 mL/min/1.7 m^2^ (IQR 3 to −6) in the amikacin group and −3 mL/min/1.7 m^2^ (IQR 2 to −8) in the non-amikacin group (*p* = 0.17). The course of eGFR is displayed in the Supplement, Figure S5. The maximum decline in eGFR from baseline within seven days was significantly higher in the amikacin group than in the non-amikacin group (−4 mL/min/1.7 m^2^ (IQR −8 to −2) vs. −2 mL/min/1.7 m^2^ (IQR −7 to 1), *p* = 0.001) (Figure 2).

Similar results were obtained when evaluating the maximum relative decline in eGFR from baseline to seven days (−4.4% (IQR 8.6 to −1.9) vs. −2.0% (IQR −6.5 to 1.1), *p* = 0.001). The median eGFR at day 90 was 83 mL/min/1.7 m^2^ (IQR 72 to 97, n = 86) in the amikacin group and 79 mL/min/1.7 m^2^ (IQR 69 to 89, n = 36) in the non-amikacin group.

The incidence of AKI increased to 8.0% (n = 10) in the amikacin group and to 4.8% (n = 6) in the non-amikacin group when a time period of 14 days after the initiation of antibiotic treatment was assessed (*p* = 0.439). Out of the seven additional AKIs, six were grade 1 and one was grade 2 (amikacin group).

### 2.3. Appropriateness of Amikacin Treatment and Concomitant Nephrotoxic Drugs

Cefepime was the beta-lactam most frequently administered in both groups (n = 237/250, 94.8%) (Table 2).

In the amikacin group, the majority of patients received amikacin for three days (n = 66/125, 52.8%, number of administrations = 292), with a daily dose of 1000 mg (n = 238/292, 81.5%) corresponding to a median daily amikacin dose of 13.7 mg per kg body weight. Overall, 55% of the patients (n = 69/125) received an inappropriate amikacin dose on the first day of administration. In 45/69 (65%) patients, the amikacin dose was lower than recommended. The appropriateness of amikacin dosing was highest in normal-weighted patients (BMI 18.5–24.9 kg/m^2^) with 55.2% (37/67), followed by overweighed patients (BMI 25–30 kg/m^2^) in 38.7% (n = 12/31). In patients with underweight (BMI < 18.5 kg/m^2^) and in obese patients (BMI > 30 kg/m^2^), the appropriateness of amikacin dosing was lower, with 33.3% (n = 2/6) and 20.0% (n = 3/15), respectively. Underweight patients were at risk of overdosing (in 50% of the patients (n = 3/6)), whereas obese patients were at risk of underdosing (in 66% of the patients (n = 10/15)).

The number of concomitant nephrotoxic drugs, such as NSAIDs or vancomycin, was higher in the amikacin group compared to the non-amikacin group (27 vs. 0, *p* < 0.001). However, diuretics were more frequently administered in the non-amikacin group compared to the amikacin group (119 vs. 47, *p* < 0.001).

## 3. Discussion

This retrospective, two-centre study demonstrates that adding amikacin for up to three days to empiric beta-lactam antibiotic therapy in autologous HSCT patients was not associated with an increased incidence of AKI. We detected an overall AKI incidence of 3.6% within seven days and 6.4% within 14 days after the initiation of antibiotic treatment, without any difference according to adjunctive amikacin administration. However, we observed a significantly larger maximum decline in eGFR within seven days after amikacin administration, although the median difference of 4 mL/min/1.7 m^2^ is small and probably negligible in this patient population. Even though the most common underlying disease in our patient population was multiple myeloma, known to be associated with impaired renal function, individuals in both centres had a high baseline eGFR (median 98 mL/min/1.7 m^2^). Only 8.4% suffered from chronic kidney disease (eGFR < 60 mL/min/1.7 m^2^) prior to empirical antibiotic treatment.

In a Cochrane analysis, which investigated the effectiveness of beta-lactam versus beta-lactam-aminoglycoside combination therapy in cancer patients with neutropenia, combination therapy was associated with an increased risk of acute renal failure (RR 0.45, 95% Confidence interval 0.35 to 0.57) [19]. However, the treatment duration of the aminoglycoside was most commonly nine days, compared to a maximum of three days in our study. Treatment duration is a known risk factor for nephrotoxicity [17,20], and reducing the treatment duration to less than three days may be associated with a decreased risk [21]. Accordingly, a Japanese randomised controlled trial assessed the effectiveness of cefepime monotherapy versus dual therapy with an adjunctive aminoglycoside for three to seven days in patients with FN and underlying haematological malignancy (mainly acute leukemia), and neither found any difference in effectiveness nor in adverse events, including renal dysfunction, which only occurred in one patient [22].

Acute and chronic renal injury are complications of autologous HSCTs due to, e.g., renal hypoperfusion or nephrotoxic drugs [23]. The latter is especially known as a risk factor for the development of AKI [17,21,23]. Two recently published Spanish studies, which assessed AKI in patients with multiple myeloma and lymphoma after autologous HSCT, reported a cumulative AKI incidence of 47.8% and 63.7% respectively [24,25]. This large difference compared to our study findings may be partly explained by the different AKI assessment approaches used in these studies. In addition to the KDIGO criteria [26], urinary output [24,25], proteinuria [23], and a much longer time span (100 days) were used to assess the occurrence of AKI. However, these studies did not compare study populations with different antibiotic regimens after autologous HSCT but focused on the overall incidence of AKI after autologous HSCT in patients with multiple myeloma [24] or lymphoma [25]. The lower incidence of AKI could also be due to the inappropriateness of the amikacin dosing in the present study, as we found that the majority of overweight and obese patients did receive a lower-than-recommended amikacin dose. This may be explained by the fear of causing AKI when a higher-than-commonly-used dose is administered.

In the present study, patients in the non-amikacin group received significantly more diuretics compared to the amikacin group, which, on the other hand, received more potentially nephrotoxic drugs other than amikacin, such as vancomycin, amphotericin, or NSAIDs, within one week prior to the initiation of empirical antibiotic treatment up to seven days after its start. However, we did not observe any difference in the incidence of AKI between the groups, which is surprising given the additive nephrotoxic effect of the latter drugs. Besides nephrotoxic drugs, we identified older age as a risk factor for AKI, which is in line with results from previous studies [15,21].

The percentage of patients with microbiologically defined infections was similar compared to a study with patients suffering from multiple myeloma (35.2% and 37.9%, respectively) [27]. However, in our study, a higher rate of bloodstream infection was detected (26.8% vs. 18.9%), mainly caused by *Escherichia coli* (*E. coli*) and *Klebsiella pneumoniae* (*K. pneumoniae*). The de-escalation treatment strategy includes the use of antibiotics that cover infections with resistant strains such as ESBL-producing pathogens during empirical treatment. However, Switzerland is a low-prevalence country in regard to ESBL-producing organisms, i.e., 10% in *E. coli* and 14% in *K. pneumoniae*, respectively [28]. Hence, in the present study, only two patients suffered from invasive infection caused by ESBL-producing pathogens, and colonisation with ESBL producing strains was only present in five patients. Three of the seven isolates were tested as being susceptible to amikacin, whereas the susceptibility pattern of the other four strains was not available. Therefore, an escalation strategy might be more appropriate for the majority of patients with FN after autologous HSCT in our setting, with few exceptions, including patients known to be colonised with resistant organisms or with septic shock. This is underscored by a Cochrane analysis encouraging the consideration of the local epidemiology in the selection of the optimal empirical treatment [19].

The strengths of our study include the inclusion of two centres and the focus on a specific and homogeneous patient population of haematological patients after autologous HSCT.

However, this study has several limitations, including the retrospective design of the study. Additionally, the baseline eGFR was high in both groups, and we were not able to consider the impact of adjunctive amikacin on the renal function in patients with chronic kidney disease. Additional factors causing AKI, such as the occurrence of septic shock, were not considered. In clinical practice, treating physicians may have decided to give an anti-pseudomonal beta-lactam monotherapy instead of a combination therapy with amikacin due to the known nephrotoxic side effects of amikacin, which may have caused a selection bias. Furthermore, the incidence of AKI was much lower than anticipated when calculating the required sample size. Hence, we cannot exclude a smaller difference in the primary endpoint, and we were not able to conduct multivariate analyses.

## 4. Materials and Methods

### 4.1. Study Design

This two-centre study used patient data from the University Hospital Basel (amikacin group) and University Hospital Bern (non-amikacin group). It was approved by both the Ethics Committee of Northwest and Central Switzerland and the Ethics Committee of Bern (BASEC No. 2023-01879). According to national regulations, subjects who had refused the written hospital’s general research consent for the use of their routinely collected data for research purposes were excluded from the study.

### 4.2. Study Population

Patients aged ≥ 18 years who underwent autologous HSCT for a haematological disorder between the 1 January 2016 and the 31 December 2022 and received an empirical antibiotic treatment consisting of a combination therapy with a beta-lactam antibiotic plus amikacin (amikacin group) or a monotherapy with a broad-spectrum beta-lactam antibiotic (non-amikacin group) within 30 days after autologous HSCT were included. Exclusion criteria included haemodialysis, outpatients, the administration of an aminoglycoside within 14 days prior (both groups) or 14 days after (non-amikacin group) the initiation of an empirical broad-spectrum beta-lactam treatment, and the administration of amikacin for more than three days (amikacin group).

### 4.3. Empirical Antibiotic Treatment

The choice of empirical antibiotic treatment was made according to the in-house recommendations of the respective centre. All patients received a broad-spectrum beta-lactam such as cefepime, piperacillin/tazobactam, meropenem, or imipenem. However, only patients being treated at the University Hospital Basel (amikacin group) additionally received the aminoglycoside amikacin once daily for one to three days. Internal guidelines recommend amikacin treatment until the return of negative blood cultures or the identification of a pathogen not requiring treatment with an aminoglycoside. The recommended amikacin doses were 15 mg/kg of body weight/24 h if the estimated glomerular filtration rate (eGFR) was >70 mL/min/1.7 m^2^, 12 mg/kg of body weight/24 h if the eGFR was 50−70 mL/min/1.7 m^2^, 7.5 mg/kg of body weight/24 h if the eGFR was 30−50 mL/min/1.7 m^2^, 4 mg/kg of body weight/24 h if the eGFR was 10−30 mL/min/1.7 m^2^, and 4 mg/kg of body weight/48 h if the eGFR was <10 mL/min/1.7 m^2^.

### 4.4. Definitions

Fever was defined as a tympanic temperature of ≥38.3 °C or ≥38.0 °C (measured twice within one hour) and neutropenia was defined as a neutrophil count <500 G/L or <1000 G/L with expected decrease. Infections were categorised into three groups: microbiologically defined infection, clinically defined infection, or fever syndrome with unknown focus [29]. The Charlson Comorbidity Index was calculated according to Charlson et al. [30]. The Chronic Kidney Disease Epidemiology Collaboration (CKD-EPI) Creatinine Equation was used to calculate eGFR in both centres. Creatinine and eGFR were evaluated at baseline (start of empirical antibiotic treatment) daily up to day 14, after 28 days (+/−10 days), and after 90 days (+/−30 days). AKI was classified according to the Kidney Disease: Improving Global Outcomes (KDIGO) criteria [26]. To assess the recovery of renal function, the lowest plasma creatinine value 14 to 90 days following the baseline creatinine was used. Recovery was achieved if creatinine was less than 50% higher compared to the baseline creatinine and if the absolute difference between the values was less than 26.5 µmol/L [31].

Concomitant treatment with nephrotoxic drugs was defined as treatment with non-steroidal anti-inflammatory drugs (NSAIDs), intravenous vancomycin, colistin or amphotericin B, torasemide, and furosemide from one week prior to the initiation of empirical antibiotic treatment up to seven days after its start.

Extended spectrum beta-lactamase (ESBL) colonisation was defined as the identification of an ESBL-producing pathogen within the previous three years from a clinically relevant specimen or screening swab. Antibiotic susceptibility testing was performed using the semi-automated VITEK2 system (bioMérieux, Petit Lancy, Switzerland).

### 4.5. Data Source

Clinical, microbiological, laboratory, and hospital-related data of included patients were retrieved from the electronic hospital information system and the medical records of the University Hospitals Basel and Bern. Study data were collected and managed using REDCap electronic data capture tools hosted at the University of Basel [32,33].

### 4.6. Outcome

The primary outcome was the incidence of AKI within seven days after the initiation of empirical antibiotic treatment. Secondary outcomes included the incidence of AKI within 14 days, the recovery of renal function after AKI, the identification of risk factors for AKI, the microbiological assessment of causative pathogens, the course of eGFR, the appropriateness of amikacin dosing, and 90-day all-cause mortality.

The appropriateness of amikacin prescriptions was assessed according to the in-house recommendations of the University Hospital Basel (amikacin group). The standard doses were 15 mg/kg of body weight/24 h if the eGFR was >70 mL/min/1.7 m^2^, 12 mg/kg of body weight/24 h if the eGFR was 50–70 mL/min/1.7 m^2^, 7.5 mg/kg of body weight/24 h if the eGFR was 30–50 mL/min/1.7 m^2^, and 4 mg/kg of body weight/24 h if the eGFR was 10–30 mL/min/1.7 m^2^. A deviation of +/−10% was accepted for the assessment of appropriateness. The adjusted body weight was used to calculate the appropriate dose [34]. If the adjusted body weight could not be determined, the total body weight was used to assess appropriate dosing.

### 4.7. Statistical Analyses

Based on the former AKI rates after autologous HSCT, a sample size of 250 patients (125 patients per study centre) was calculated to detect a relative difference of 60% with a power of 80% and an alpha of 0.05 in the primary outcome. Consequently, 125 patients per centre hospitalised between 1 January 2016 and 31 December 2022 were randomly selected for analysis. Continuous variables were summarised using the median and interquartile range (IQR), and categorical variables were summarised using counts and frequencies. To assess differences between the two centres, χ2 tests were used for categorical data, and Mann–Whitney U tests were used for continuous non-normally distributed variables. The normality of distribution was tested using the Kolmogorov–Smirnov test. A two-sided *p*-value < 0.05 was considered statistically significant. Univariate analysis was performed to identify factors associated with the primary outcome. Due to the low number of events, no multivariate analysis was performed. The cumulative incidence of AKI according to amikacin treatment was calculated by the Kaplan–Meier test and compared by the log rank test. All analyses and figures were performed using SPSS Version 28 (IBM SPSS Statistics for Windows. Armonk, NY, USA), R statistical software (version 4.2.1), and GraphPad (version 9; GraphPad Software, San Diego, CA, USA).

## 5. Conclusions

In summary, the administration of amikacin up to three days was not associated with an increased incidence of AKI after autologous HSCT in individuals without evidence of pre-existent chronic renal failure. We conclude that the short-term administration of amikacin in FN in haematological autologous transplant patients with normal to high baseline eGFR values is safe regarding renal function but should be scrutinised due to a very limited benefit in a low-resistance setting.

## Figures and Tables

**Table 1:** Patient baseline characteristics.

Variable	Amikacin Group, Centre 1 (*n* = 125)	Non-Amikacin Group, Centre 2 (*n* = 125)	Total (*n* = 250)	*p*-Value
Number of patients	125	125	250	
Age (years)	61 (55–67)	62 (55–67)	61 (55–67)	0.923
Male sex	87 (70)	76 (61)	163 (65)	0.184
BMI (kg/m^2^)	24 (22–27)	24 (22–27)	24 (22–27)	0.281
Charlson Comorbidity Score	3 (2–4)	3 (2–4)	3 (2–4)	0.034
Haematological disorder				0.001
Multiple myeloma	70 (56)	67 (54)	137 (55)	
Acute leukaemia	1 (1)	13 (10)	14 (6)	
Lymphoma	54 (43)	43 (34)	97 (39)	
Amyloid light-chain-amyloidosis	0 (0)	2 (2)	2 (1)	
Laboratory parameter ^†^				
Creatinine (µmol/L)	65 (54–81)	59 (46–78)	63 (49–80)	0.057
eGFR (mL/min/1.7 m^2^)	96 (85–105)	100 (86–111)	98 (85–108)	0.060
eGFR<60 mL/min/1.7 m^2^	9 (7)	12 (10)	21 (8)	0.468
C-reactive protein (mg/L)	26 (11.0–53)	23 (12–52)	24 (11–53)	0.729
Leucocytes (G/L)	0.06 (0.04–0.10)	0.04 (0.02–0.10)	0.05 (0.03–0.10)	0.005
Neutrophils (G/L)	0.02 (0.01–0.03)	0.03 (0.01–0.18)	0.02 (0.01–0.04)	0.158
Albumin (g/L)	30 (27–32)	28 (25–29)	29 (27–31)	˂0.001
Definition of infection				0.005
Clinically defined infection	33 (26)	50 (40)	83 (33)	
Microbiologically defined infection	41 (33)	47 (38)	88 (35)	
Fever syndrome of unknown focus	51 (41)	28 (22)	79 (32)	
Infection site				0.033
Mucositis/Gastrointestinal tract	81 (65)	85 (68)	78 (67)	
Respiratory tract	7 (6)	6 (5)	13 (5)	
Urinary tract	0 (0)	6 (5)	6 (2)	
Catheter	6 (5)	1 (0.4)	7 (3)	
Central nervous system	0 (0)	1 (0.4)	1 (0.4)	
Other	31 (25)	26 (21)	57 (23)	
	34 (27)	33 (26)	67 (27)	
Bloodstream infections ^‡^				1.000
* Escherichia coli*	21 (62)	18 (55)	39 (58)	
* Escherichia coli* ESBL	0 (0)	1 (3)	1 (2)	
* Klebsiella pneumoniae*	1 (3)	6 (18)	7 (10)	
Coagulase-negative staphylococci	0 (0)	3 (9)	3 (5)	
* Pseudomonas aeruginosa*	2 (6)	3 (9)	5 (8)	
* Serratia marcescens*	1 (3)	0 (0)	1 (2)	
* Enterococcus avium*	1 (3)	0 (0)	1 (2)	
* Enterococcus faecalis*	0 (0)	1 (3)	1 (2)	
* Gemella species*	1 (3)	0 (0)	1 (2)	
Gram positive rods	1 (3)	0 (0)	1 (2)	
* Streptococcus mitis* group	6 (18)	0 (0)	6 (9)	
Viridans streptococci	0 (0)	1 (3)	1 (2)	
Outcome				
Length of stay	23 (21–26)	21 (19–23)	22 (20–25)	˂0.001
Mortality 30 days	0 (0)	1 (1)	1 (0.4)	1.000
Mortality 90 days	0 (0)	1 (1)	1 (0.4)	1.000

Data are presented as count (percentages) or median (interquartile range). ^†^ at start of amikacin treatment (centre 1) or beta-lactam antibiotic treatment (centre 2). ^‡^ percentages for bloodstream infections were calculated according to the respective total numbers (not the total number of patients). Abbreviations: BMI, body mass index; eGFR, estimated glomerular filtration rate; ESBL, extended spectrum beta-lactamase.

**Table 2:** Empirical antibiotic treatment.

Variable	Amikacin Group, Centre 1 (*n* = 125)	Non-Amikacin Group, Centre 2 (*n* = 125)	Total (*n* = 250)
Beta-lactam antibiotic			
Cefepime	118 (94)	119 (95)	237 (95)
Piperacillin/tazobactam	7(6)	2 (2)	9 (4)
Meropenem	0 (0)	4 (3)	4 (2)
Amikacin treatment			
Number of days of amikacin administration			
1 day	24 (19)		
2 days	35 (28)		
3 days	66 (53)		
Total dose of amikacin (g)	2.25 (2.0–3.0)		
Daily amikacin dose			
500–900 mg	41 (14)		
1000 mg	238 (82)		
1200–1500 mg	13 (5)		
Median daily amikacin dose (mg/kg body weight)	13.7 (12.1–14.9)		
Number of concomitant nephrotoxic drugs other than amikacin ^†^			
0	65 (52)	6 (5)	71 (28)
1	38 (30)	106 (85)	144 (58)
2	20 (16)	13 (10)	33 (13)
3	2 (2)	0 (0)	2 (1)

^†^ Concomitant nephrotoxic drugs other than amikacin included non-steroidal anti-inflammatory drugs, vancomycin, amphotericin B, or diuretics administered −7 to +7 days prior to or after the initiation of the antibiotic treatment. Data are presented as count (percentages) or median (interquartile range).

## Data Availability

The datasets used and/or analysed in the current study are available from the corresponding author upon reasonable request.

## Supplementary Materials

The following supporting information can be downloaded at: https://www.mdpi.com/article/10.3390/antibiotics14090919/s1, Figure S1: Flowchart of patients included in the study. † including missing baseline creatinine (n = 12) and discharge before day 7 (n = 29). Abbreviations: HSCT: autologous hematopoietic stem cell transplantation; Figure S2: Course of eGFR between patients who experienced acute kidney injury (yellow) and those who did not (blue). Means with standard deviation are depicted. Abbreviations: eGFR: estimated glomerular filtration rate; AKI: acute kidney injury; Figure S3: Amikacin dose administered on the first day of amikacin treatment in patients who experienced AKI and those who did not. Horizontal line: median amikacin dose. Abbreviations: AKI, acute kidney injury; ns, not statistically significant; BW, body weight; Figure S4: The difference in cumulative amikacin dose in the amikacin group in patients who experienced acute kidney injury and those who did not. Horizontal line: median cumulative amikacin dose. Abbreviations: AKI, acute kidney injury; ns, not statistically significant; Figure S5: Course of eGFR in the Amikacin and Non-Amikacin groups. Means with standard deviation are depicted. Abbreviations: eGFR, estimated glomerular filtration rate.

## Abbreviations

The following abbreviations are used in this manuscript:

AKI	acute kidney injury
CKDEC_EPI	Chronic Kidney Disease Epidemiology Collaboration
eGFR	estimated glomerular filtration rate
ESBL	extended spectrum beta-lactamase
