# Supplementary material for: Incidence of Acute Kidney Injury in Autologous Hematopoietic Stem Cell Transplant Recipients According to the Administration of Empirical Amikacin: A Two-Centre Retrospective Cohort Study
Source: Antibiotics (Basel). 2025 Sep 11;14(9):919. doi: 10.3390/antibiotics14090919 (PMC12466445; doi:10.3390/antibiotics14090919)
Supplement: Supplementary file 1 [file antibiotics-14-00919-s001.zip › antibiotics-3846626-supplementary.pdf]

**Supplement**

**Figure S1.** Flowchart of patients included in the study. †including missing baseline creatinine (n=12) and discharge before day 7 (n=29). Abbreviations: HSCT: autologous hematopoietic stem cell transplantation.

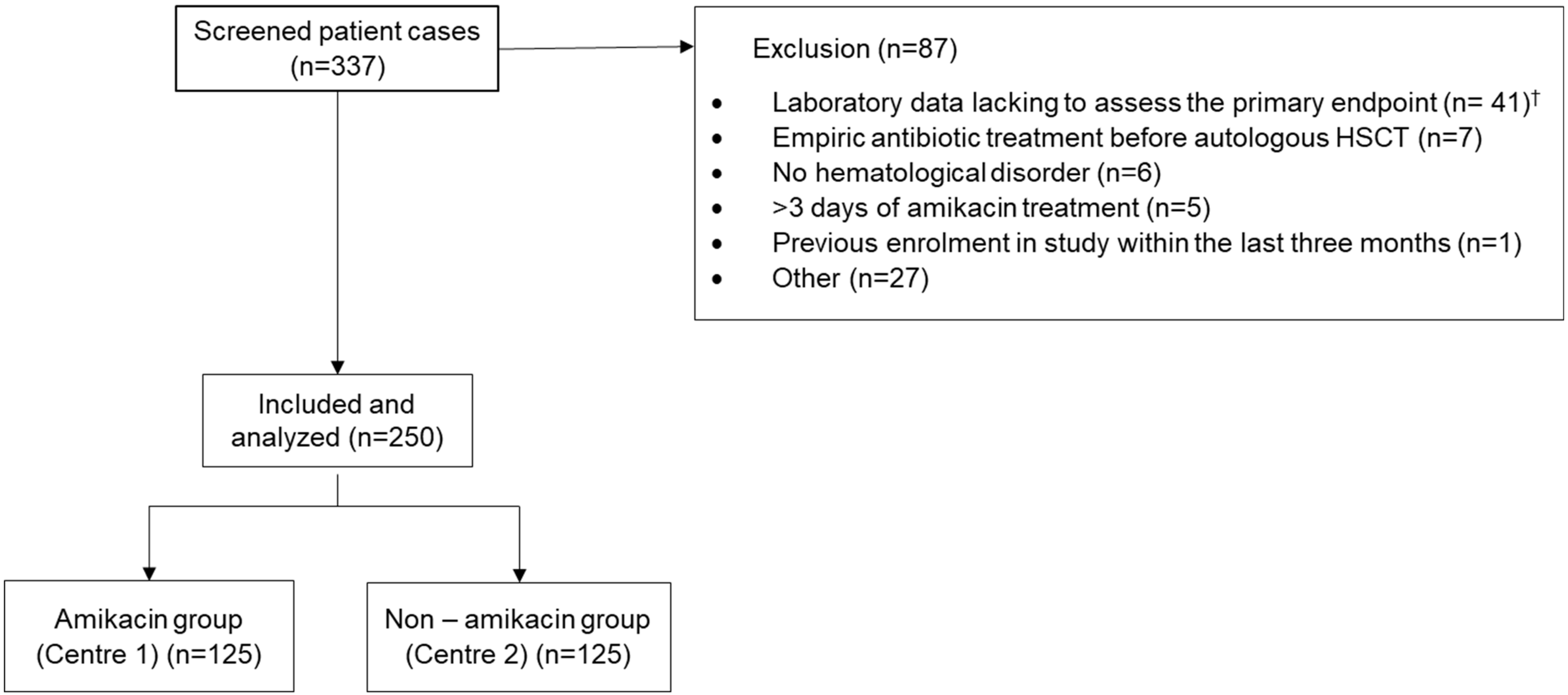

**Figure S2.** Course of eGFR between patients who experienced acute kidney injury (yellow) and those who did not (blue). Mean with standard deviation are depicted. Abbreviations: eGFR: estimated glomerular filtration rate; AKI: acute kidney injury.

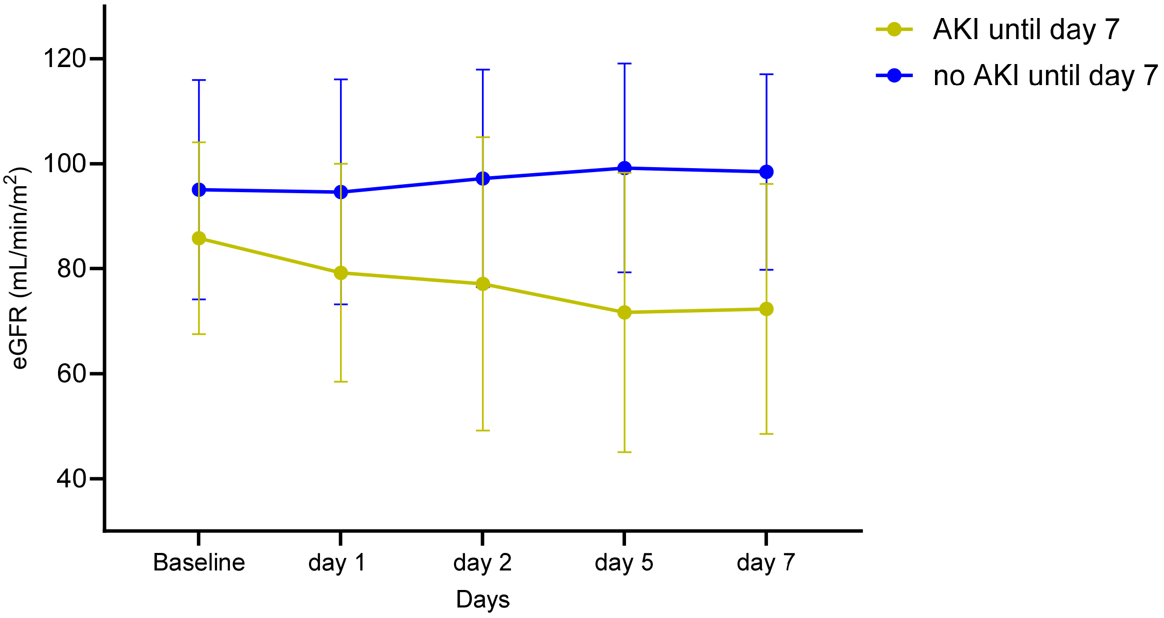

**Figure S3**. Amikacin dose administered on the first day of amikacin treatment in patients who experienced AKI and those who did not. Horizontal line: median amikacin dose. Abbreviations: AKI, acute kidney injury; ns, not statistically significant; BW, body weight.

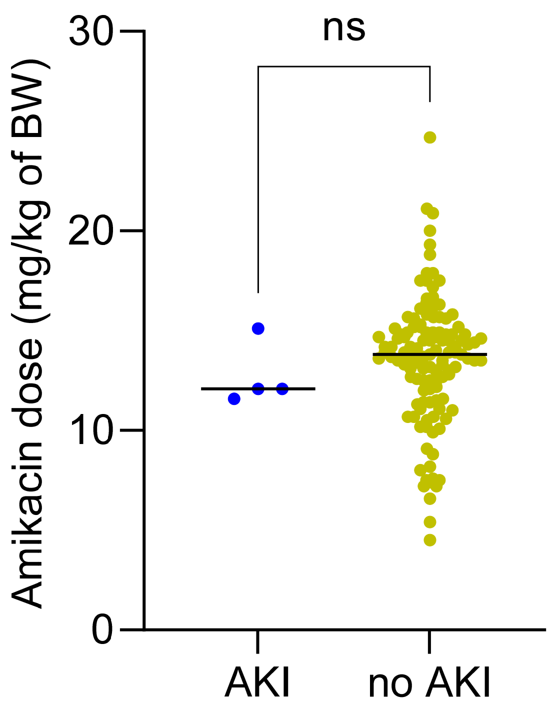

**Figure S4**. The difference in cumulative amikacin dose in the amikacin group in patients who experienced acute kidney injury and those who did not. Horizontal line: median cumulative amikacin dose. Abbreviations: AKI, acute kidney injury; ns, not statistically significant.

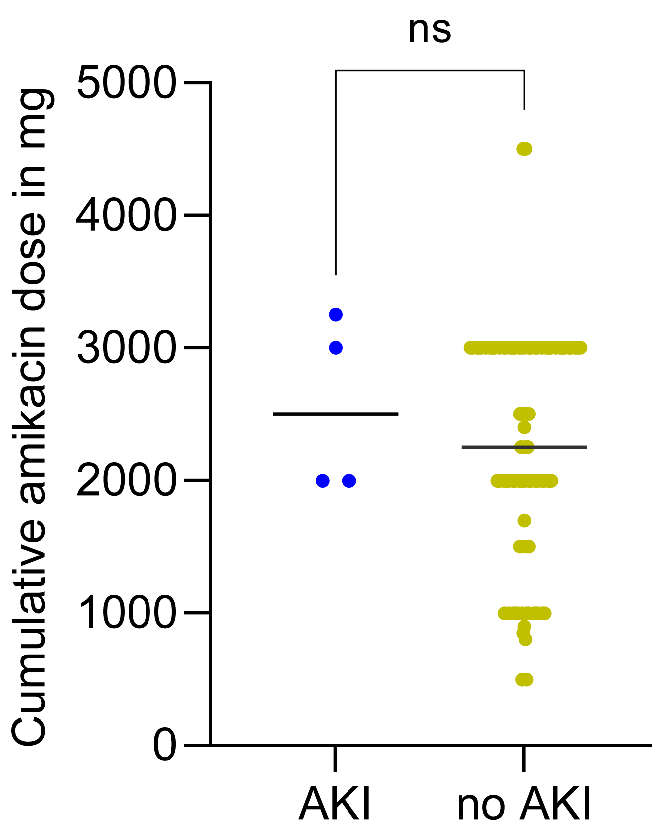

**Figure S5.** Course of eGFR in the Amikacin and the Non-amikacin group. Mean with standard deviation are depicted. Abbreviations: eGFR, estimated glomerular filtration rate.

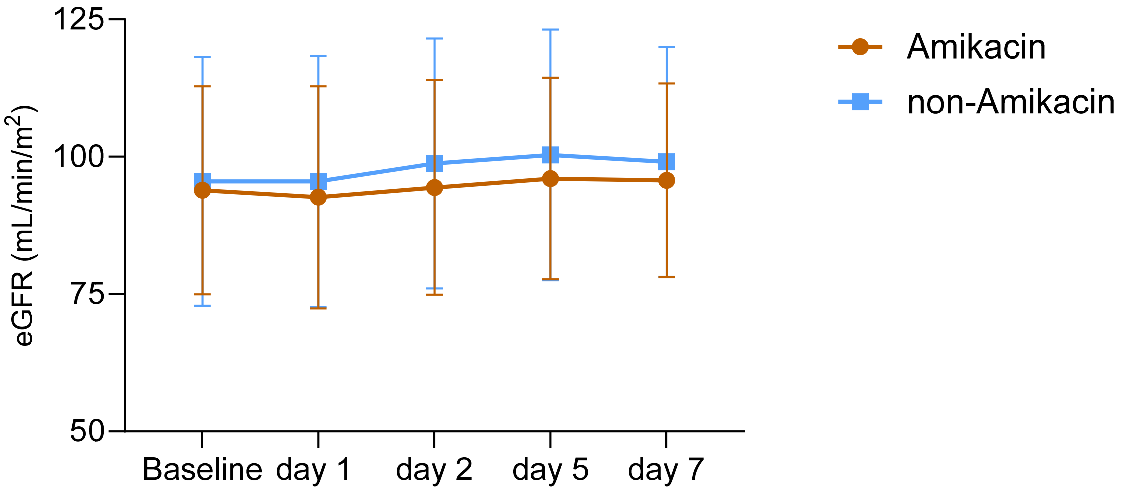


.
